# A Protocol for Comparing Dry and Wet EEG Electrodes During Sleep

**DOI:** 10.3389/fnins.2020.00586

**Published:** 2020-06-17

**Authors:** Sven Leach, Ku-young Chung, Laura Tüshaus, Reto Huber, Walter Karlen

**Affiliations:** ^1^Child Development Center and Pediatric Sleep Disorders Center, University Children’s Hospital Zurich, University of Zurich, Zurich, Switzerland; ^2^Mobile Health Systems Lab, Department of Health Sciences and Technology, Institute of Robotics and Intelligent Systems, ETH Zürich, Zurich, Switzerland; ^3^Department of Child and Adolescent Psychiatry and Psychotherapy, Psychiatric Hospital, University of Zurich, Zurich, Switzerland

**Keywords:** mobile EEG, dry electrodes, signal quality, sleep slow waves, sleep spindles, sleep staging, testing protocol, natural settings

## Abstract

**Background:**

Sleep is commonly assessed by recording the electroencephalogram (EEG) of the sleeping brain. As sleep assessments in a lab environment are cumbersome for both the participant and researcher, it would be highly desirable to record sleep EEG with a user-friendly and mobile device. Dry electrodes that are reusable, low-cost, and easy to apply would be an essential component of such a device. In this study, we developed a testing protocol to investigate the performance of novel flat-type dry electrodes for sleep EEG recordings in free-living conditions.

**Methods:**

Overnight sleep EEG, electrooculogram and electromyogram of four young and healthy participants were recorded at home. Two identical ambulatory recording devices, one using novel flat-type dry electrodes, the other using self-adhesive pre-gelled electrodes, simultaneously recorded sleep EEG. Between both electrode types, we then compared the signal quality, the incidence of artifacts, the sensitivity, specificity and inter-scoring reliability (Cohen’s kappa) of sleep staging, as well as the agreement of important characteristics of sleep-specific EEG microstructure features, such as slow waves (0.5–4 Hz) and sleep spindles (10–16 Hz).

**Results:**

Our testing protocol comprehensively compared the two electrode types on a macro- and microstructure level of sleep. The dry and pre-gelled electrodes both had comparable signal quality and sleep staging was feasible with both electrodes. Also, slow-wave and spindle characteristics were similar. However, sweat artifacts were more prevalent in the flat-type dry electrodes.

**Conclusion:**

With a reliable testing protocol, the performance of dry electrodes can be compared to reference technologies and objectively assessed also in free-living conditions.

## Introduction

The benefits of sleep on physical and mental health are evident. However, getting a restful night of sleep can be a difficult endeavor. The quality of sleep is a critical parameter for the restfulness of sleep. Current wearable sleep technologies, such as smartwatches and wrist-, arm-, and headbands, aim at assessing sleep quality by providing details of the macrostructure of sleep, i.e., the temporal organization of the night into sleep stages. However, sleep quality depends on both, the macro- and microstructure of sleep, the latter being the identification and quantification of sleep-specific neurophysiological events (Malinowska et al., 2006).

While the macrostructure of sleep can, to a limited extent, be monitored with movement-based wearables and plotted in a hypnogram on a timescale of hours, detailed information about the macro- and microstructure can only be obtained by recording the electroencephalogram (EEG) of the sleeping brain. Consequently, the EEG is part of every scientific and clinical assessment of sleep. However, this assessment typically involves lab visits, stationary amplifiers, as well as a challenge for participants to sleep in an unfamiliar environment, which can lead to the well-known first night effect (Toussaint et al., 1997). This first night effect together with a considerable night-to-night variability of sleep, even under controlled conditions (Buckelmüller et al., 2006), limits the validity of a single night assessment in a research or clinical setting.

Therefore, it is highly desirable to record sleep EEG with a simple, user-friendly, and low-cost mobile device in free-living conditions for extended periods. Dry electrodes could constitute a major component of such a device, as they are reusable, low-cost, and able to establish sufficient electrical contact with the skin without the necessity of electrode gel. However, the use of alternative electrodes for sleep recordings is not evident, as the limited positioning options and altered contact properties might affect derived sleep parameters, including the sleep micro- and macrostructure. Consequently, at each introduction of novel electrode types for sleep monitoring, it is essential to test and characterize these for the specific application.

### The Importance of Assessing Sleep Structure

Even though the overall macrostructure of sleep shows high variability between nights even within the same individual, one can affirm that, night after night, the sleeping brain cycles through various sleep stages in a repetitive manner. If this peculiar sleep pattern turns abnormal, it is indicative of a variety of adverse health conditions (Luyster et al., 2012), such as coronary heart disease (Ayas et al., 2003) or obesity and diabetes mellitus type 2 (Tan et al., 2018).

The EEG is the basis for organizing a night of sleep, epoch by epoch, into different sleep stages (Iber et al., 2007). Sleep is commonly classified into rapid-eye movement (REM) sleep and non-rapid-eye movement (NREM) sleep. NREM sleep is further subdivided into sleep stages N1, N2, and N3, which reflects increasing sleep depth, i.e., N1 represents light and N3 deep sleep. During NREM sleep, distinct EEG events with typical frequencies occur: slow waves (0.5–4 Hz), K-complexes (single slow waves) and sleep spindles (10–16 Hz). The occurrence of either of these are the hallmark for sleep stage N2. Slow waves and sleep spindles continue to occur in the deepest sleep stage N3, where slow waves with a peak-to-peak amplitude of at least 75 μV dominate at least 20% of the evaluated epoch.

Slow waves and sleep spindles are of particular interest, as they are tightly linked to memory consolidation (Rasch and Born, 2013) and restorative functions (Vyazovskiy and Harris, 2013; Tononi and Cirelli, 2014). Certain characteristics of their morphology change depending on prior cognitive challenges during wake, i.e., the amplitude and slope of slow waves are increased when preceded by specific learning experiences (Huber et al., 2004; Molle et al., 2004) and decreased when the encoding of information was prevented (Huber et al., 2006). In addition, slow waves are the primary biomarker for sleep pressure, i.e., the drive to fall asleep (Dijk et al., 1993; Borbély and Achermann, 1999). Together with sleep spindles, they account for an important part of the microstructure of sleep.

Usually, the macrostructure of sleep is depicted by a hypnogram, which is determined by scoring three EEG (frontal, central, and occipital), two EOG and one chin EMG derivation. However, a single frontal electrode referenced to the contralateral mastoid alone can capture a large proportion of ongoing neurophysiological events during sleep, as slow waves are most pronounced over frontal areas in both younger and older adults (Landolt and Borbély, 2001), and as sleep spindles are typically found over fronto-central areas (Cox et al., 2017). However, alpha activity (spectral power between 8 and 12 Hz), an important marker for the onset of sleep stage N1, is most pronounced in occipital electrodes. Accordingly, when a scoring based on a single frontal EEG derivation is compared to a three derivation scoring, the agreement of N2 and N3 is high, whereas the agreement of N1 is lower.

### Type of Electrodes Used in Wearable EEG Systems

In order to obtain a high-quality EEG with a wearable device, a substantial requirement is the use of high performance electrodes. The electrodes need to ensure a good and constant electrical contact with the skin and therefore need low impedance properties. The electrode-skin contact can either be ensured by adding a conductive gel between the electrode and the skin or by using a conductive material with a high contact surface that ensures electrical contact.

Pre-gelled electrodes have previously been used in wearable EEG systems to measure overnight sleep EEG with high signal quality, but a replacement after each measurement is necessary, rendering them not economical in case of prolonged use. Therefore, replacing pre-gelled electrodes with re-usable dry electrodes at fixed positions in an integrated device would reduce costs and improve usability of wearable EEG systems.

EEG measurements can be performed with different types of re-usable dry electrodes. Pin-type electrodes are designed to reach the scalp through dense hair, but because they are not attached to the skin directly, they have the disadvantage of being subject to strong motion artifacts and can change position throughout the night (Li et al., 2016). Therefore, they require high pressure on the scalp to ensure high signal quality which causes discomfort and even pain during prolonged use (Gao et al., 2018). Bristle electrodes with softer pins are perceived more comfortable, but still require high contact pressure, especially after long-term use without recoating (Grozea et al., 2011). Unlike the pin-type electrodes, flat types do not cause pain or discomfort as they are soft, bendable, and ensure low contact impedance with a high contact surface. On the downside, due to their larger size, they require hair-free or prepared skin to create sufficient contact to the skin. This limits their application to frontal (i.e., on the forehead) electrode positions.

### Methods to Evaluate Electrodes Used in Wearable EEG Systems

To enable sleep scoring and analysis, dry electrodes should be sufficiently robust to artifacts that might occur during sleep (e.g., movement and sweating) and not introduce any additional interferences. Furthermore, they need to offer electrical and physical properties that enable the recording of important sleep characteristics such as slow waves and spindles. In general, EEG electrodes should have low skin contact impedance to prevent signal attenuation and impedance mismatch, the main cause for ineffective common mode rejection, i.e., the ability of the differential amplifier to cancel out the signals that are common to both electrodes (Ferree et al., 2001). Particular to wearable applications, the electrode specifications should include a certain level of tolerance toward imperfect placement by inexperienced users that could cause additional artifacts.

The importance of assessing data quality of wearable EEG systems has been identified, but only few studies exist that examine signal quality with respect to electrodes (Radüntz, 2018) and no standardized methodology is available (Casson, 2019). Previous work that evaluated the suitability of electrodes specifically for sleep applications strongly focuses on comparing the macrostructure of the derived sleep patterns. For example, characteristics necessary to perform sleep scoring are evaluated to test a novel dry electrode array around the ear (Sterr et al., 2018). Of main interest are the Bland-Altmann agreement and Pearson correlations of macrostructure parameters obtained after scoring such as the duration of sleep stages. In addition, parameters are compared epoch-by-epoch (Griessenberger et al., 2013; Sterr et al., 2018). In a feasibility study of a tattoo-based electrode setup for sleep, four nights were recorded at the subjects’ home, and sleep is scored by an expert to qualitatively evaluate the EEG and to visually determine whether typical sleep patterns (e.g., spindles and slow waves) can be distinguished (Shustak et al., 2019). Introducing additional quantitative measures, Ferster et al. (2019) compare the correlation of the mean square power in the delta (0.5–4 Hz) and sigma (10–15 Hz) bands during NREM sleep. This comparison uses two separate portable amplifiers that are designed for home-based sleep screening, of which the reference system is a clinically established device. The challenge of using two completely separate systems is the time synchronization between the amplifiers, which leads to only visual and qualitative comparisons or large comparison windows (Ferster et al., 2019). More often, comparative electrode studies rely on a single amplifier system that shares a common reference (and ground) of either electrode type, which enables correlation analysis in the time domain, but may introduce unwanted distortions in the opposite channel (Casson, 2019). Sequential testing of different single-type electrode configurations is not possible in physiological monitoring due to the strong time dependency of the signal. Even in lab-based studies, the quantitative assessment of sleep microstructure is rarely evaluated during electrode testing. Furthermore, differences between various EEG systems and electrodes can also be matched to factors other than technological variability, such as subject and session variability (Melnik et al., 2017). Therefore, it is essential to control for these effects in electrode comparisons. To our best knowledge, the evaluation of EEG electrode quality outside of controlled laboratory conditions and comprising a detailed analysis of sleep micro- and macrostructure as well as a comparison with a reference electrode type has not yet been reported. In summary, no established methodology exists to objectively evaluate and compare electrodes for sleep applications.

Our aim was to establish a reproducible electrode testing protocol that would enable the comparison of essential features to characterize the macro- and microstructure of sleep and highlight differences and limitations that occur when used in a natural setting. In particular, we evaluated the suitability of electrodes for scoring sleep from at home overnight recordings. Furthermore, we investigated the signal quality and the sensitivity to artifacts to evaluate whether the electrodes would be reliable enough for unsupervised recordings of sleep EEG.

## Materials and Methods

We developed the testing protocol by following a realistic procedure where novel flat-type dry electrodes are compared against established pre-gelled electrodes. This included the design of an amplifier setup that would enable simultaneous recording of sleep EEG using two types of electrodes in a natural setting, the conduction of such data collection, and the development of the analysis and evaluation parameters that include relevant sleep macro- and microstructure metrics.

### Electrodes

**FIGURE 1:**
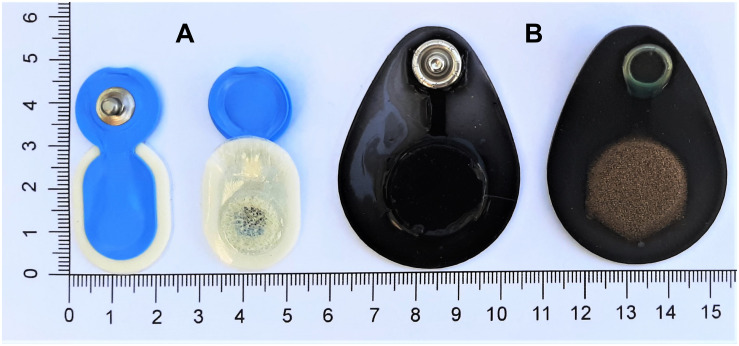
Evaluated electrodes. (**A**, left) Ambu^®^ Neuroline 720 pre-gelled electrodes (Ambu A/S, DK) and **(B**, right) Dryode^TM^ electrodes (IDUN Technologies, CH).

We evaluated the performance of a novel generation of dry, flat-type electrodes (Dr) and self-adhesive pre-gelled (Pg) electrodes. The Dryode^TM^ electrodes (IDUN Technologies, CH, Figure 1B) featured a combination of conductive textiles and polymers. They consisted of a knitted silver-coated nylon fabric with a sensor area of 18–20 mm^2^. Pg electrodes (Ambu^®^ Neuroline 720-00-S, Ambu A/S, DK, Figure 1A) were disposable and specifically marketed for sleep EEG. They featured Ag/AgCl sensor material with a sensor area of 18 mm^2^ and a gel area of 95 mm^2^.

The Dr electrode design optimizes adhesion to the skin which reduces noise artifacts (Stauffer et al., 2018). We considered this electrode particularly interesting for sleep monitoring applications because of the skin-contact impedance below 50 kΩ⋅cm^2^ (Stauffer et al., 2018). Furthermore, the bendable design enabled electrode attachment on curved locations (e.g., mastoids) and measurements of long duration would be possible with high comfort and no skin irritations (Stauffer et al., 2018). However, these electrodes have not been validated for use in overnight sleep studies to date and were therefore of interest for an electrode comparison.

### Data Collection

#### Experiments

We designed our experiments to gather simultaneous recordings from Dr and Pg electrodes under identical conditions during overnight sleep. The experiments were conceptualized with a realistic environment at home in mind. The study was conducted in accordance with the Declaration of Helsinki and approved by the institutional ethics committee (ETH EK 2017-N-67).

**FIGURE 2:**
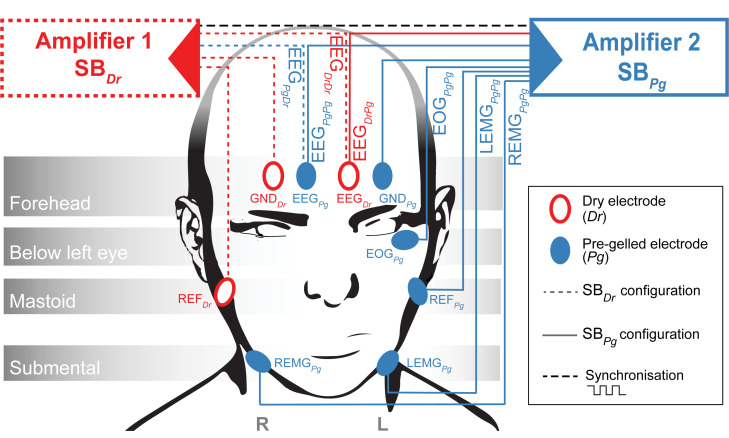
Amplifier Setup. Amplifier 1 (Red, SB_Dr_) measures EEG_DrDr_ and EEG_PgDr_ while referenced (REF_Dr_) and grounded (GND_Dr_) to Dr electrodes. Amplifier 2 (Blue, SB_Pg_) measures EEG_DrPg_, EEG_PgPg_, EOG_PgPg_, LEMG_PgPg_ and REMG_PgPg_ while referenced (REF_Pg_) and grounded (GND_Pg_) to Pg electrodes. A trigger signal is exchanged between the two amplifiers to synchronize the sampling.

Two identical MHSL-SleepBand (SB) biosignal amplifiers featuring a high-end 8-channel 24-bit analog-to-digital converter (ADS1299, Texas Instruments Inc., United States) were set up to measure EEG (Ferster et al., 2019). Each SB was powered with lithium batteries (2600 mAh, 3.63 V, 9.5 Wh). The SB is a mobile sleep monitoring system that provides research quality EEG recordings and on-board real-time processing specifically designed for sleep research. The amplifiers were set up with two different electrode configurations (SB_Dr_ or SB_Pg_, Figure 2). SB_Dr_ (Amplifier 1) was referenced and grounded to Dr electrodes (REF_Dr_ and GND_Dr_), whereas SB_Pg_ (Amplifier 2) was referenced and grounded to Pg electrodes (REF_Pg_ and GND_Pg_). Both amplifiers recorded a common 1 Hz synchronization signal. For EEG recordings, the Dr electrode was placed on the right forehead (corresponding to Fp2, EEG_Dr_), whereas the Pg electrode was placed on the left forehead (corresponding to Fp1, EEG_Pg_). The reference electrodes (REF_Dr_ and REF_Pg_) were placed on the contralateral, the ground electrodes (GND_Dr_ and GND_Pg_) on the ipsilateral mastoid with respect to the respective frontal electrode. Both devices simultaneously measured EEG_Dr_ and EEG_Pg_ using splitters. SB_Dr_ measured EEG_Dr_ and EEG_Pg_ referenced to REF_Dr_ and grounded to GND_Dr_, resulting in the EEG_DrDr_ and EEG_PgDr_ derivations (the derivation subscript represents the electrode type used to obtain the EEG followed by the reference/ground electrode type). SB_Pg_ measured EEG_Dr_ and EEG_Pg_ referenced to REF_Pg_ and grounded to GND_Pg_, resulting in the EEG_DrPg_ and EEG_PgPg_ derivations. Consequently, EEG_DrDr_ and EEG_PgPg_ refer to an EEG derivation entirely based on Dr and Pg electrodes, respectively. With EEG_DrPg_ and EEG_PgDr_, we disentangled the EEG and reference electrode, which allowed to study whether the EEG or reference electrode was responsible for a potentially bad signal. We compared EEG_DrDr_ against EEG_PgPg_, since EEG_PgPg_ has been successfully used to acquire overnight sleep EEG using the same SB-electrode configuration and showed non-inferiority to a certified system (Ferster et al., 2019). Additionally, SB_Pg_ recorded the electrooculogram (EOG_PgPg_), and the left and right electromyogram (LEMG_PgPg_ and REMG_PgPg_) derivations. In the proposed analysis, EOG and EMG signals were not further investigated. All channels were recorded with a sampling frequency of 250 Hz. The set of Dr electrodes was re-used and cleaned with alcohol wipes after each recording. A fresh set of Pg electrodes was applied for each new recording.

#### Participants

After written informed consent, overnight EEG, EOG, and EMG were recorded from five young, healthy participants (age range: 25.2–30.0 years, 3 male, 1 left handed, 1 ambidextrous) at their homes. Participants self-reported no health and sleep problems, neurological, psychiatric or internal disorders, skin conditions, skin allergies, or recent drug consumption. All participants had a normal BMI (range: 20.7–25.2) and a habitual caffeine consumption of 0–7 cups of coffee or energy drinks per day, as well as 0–2 cups of black tea, green tea or caffeinated lemonade per day. Habitual sleep times (weekday range: 11:00 p.m.–01:00 a.m., weekend range: 11:00 p.m.–03:00 a.m.) and sleep duration (weekday range: 6–8 h, weekend range: 7.5–10 h) were collected to time the experiment according to habitual bed times. One day before the experiment, we asked participants to refrain from alcohol, and excessive caffeine and nicotine consumption to ensure normal skin conditions, body temperature, and sweat production during the following night. We asked participants to go to bed at their usual bedtime and avoid late-night activities the night before the experiment. After the experimenter attached the electrodes, the signal quality and contact impedance was visually verified in a graphical user interface. Despite the supervised electrode attachment, one recording had to be discarded due to poor attachment of the reference dry electrode on the mastoid.

### EEG Analysis

We conducted an in-depth evaluation of the macro- and microstructure of sleep recorded from both Dr and Pg electrodes. We conducted the following analyses to validate the potential of both electrodes to be used in sleep research: (1) performance in sleep scoring, (2) visual inspection of detected artifacts, (3) capability to detect important sleep characteristics such as slow waves and sleep spindles. We further examined the frequency domain to test whether the electrodes are capable of measuring sleep EEG signals and whether the signal quality and spectral response agree between electrodes.

#### Pre-processing

The data collected from both SB_Dr_ and SB_Pg_ were time-synchronized with linear interpolation using the commonly recorded markers at the beginning and end of the experiments and the 1 Hz synchronization signal. Biosignals were converted to μV, notch-filtered to remove 50 Hz power-grid noise, band-pass filtered to the frequency of interest, and segmented into 20 s epochs. The cut-off frequencies for the respective band-pass filter were dependent on the type of analysis and are reported below. The MATLAB code for filtering is reported in the Supplementary Material. The power spectral density (PSD) was calculated for each epoch on EEG data that were band-pass filtered between 0.5 and 40 Hz using the Welch method (4 s Hanning windows, resolution 0.25 Hz).

#### Sleep Scoring

To assess whether the EEG signal from Dr electrodes is suitable for sleep scoring, we compared the two single-derivation scorings against each other. For this purpose, the 8 single-derivation EEG signals (EEG_DrDr_ and EEG_PgPg_ of each participant) were band-pass filtered between 0.5 and 40 Hz, randomized, and presented to a single sleep expert who was blinded to the signal’s origin (type of electrode and participant). Sleep stages were scored epoch by epoch based on standard criteria (Iber et al., 2007; Berry et al., 2017) except for the inclusion of only a single frontal EEG derivation. Sleep scoring was performed using a software obtained from the Institute of Pharmacology and Toxicology of the University of Zurich. To avoid the bias of inter-rater variability in the comparison of the scoring between the two types of electrodes, all recordings were scored by a single expert.

#### Artifact Identification

**FIGURE 3:**
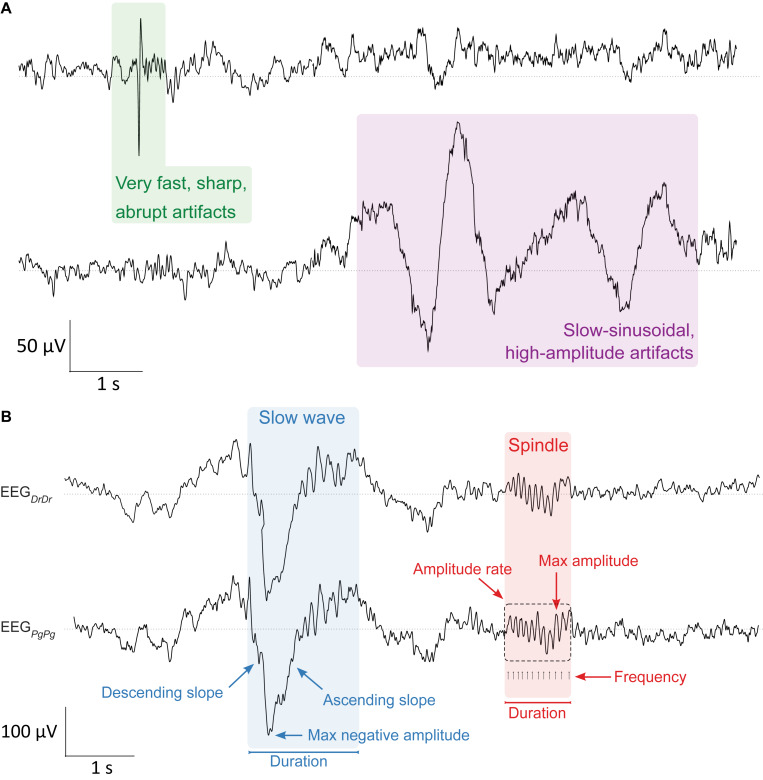
Artifact and sleep microstructure characteristics. (**A**, top) Two types of artifacts were visually identified in the EEG during sleep scoring. Very fast, sharp, abrupt artifacts (green, left) and slow-sinusoidal, high-amplitude artifacts (purple, right). They were considered as artifacts and not slow waves whenever they were only present in one EEG derivation. (**B**, bottom) Characterization of major properties of slow waves (0.5–4.0 Hz, blue) and sleep spindles (10–16 Hz, red) that were essential for determining sleep quality in the EEG signal. Slow waves were characterized by their maximum negative amplitude (μV), duration (s), and the descending and ascending slope (μV/s), the maximum steepness of slow waves either between the negative zero-crossing and the maximum negative amplitude, or the maximum negative amplitude and the positive zero-crossing, respectively. Spindles were characterized by their maximum amplitude (μV), duration (s), frequency (Hz), and amplitude rate (μV/s), the sum of all absolute data points over the duration of spindles. Slow waves and sleep spindles looked similar in both electrode types EEG_DrDr_ (top) and EEG_PgPg_ (bottom).

During sleep scoring, the expert additionally visually identified and marked 4 s windows containing artifacts in either or all of the four EEG derivations (EEG_DrDr_, EEG_Pg__Dr_, EEG_PgPg_, EEG_Dr__Pg_). While doing so, he quantified two distinct types of artifacts: very fast, sharp, abrupt artifacts and slow-sinusoidal, high-amplitude artifacts (Figure 3A). Afterward, we used in addition a semi-automatic artifact detection algorithm (Huber et al., 2000), which, in all four EEG derivations separately, marked 20 s epochs whose power exceeded a threshold defined by the average power value in the 0.75–4.5 Hz and the 20–30 Hz band in sleep scored N1, N2, and N3 epochs.

#### Analysis of EEG Artifacts

The total number and ratio of epochs containing artifacts for each single derivation were reported. The occurrence numbers of very fast, sharp, abrupt artifacts and slow-sinusoidal, high-amplitude artifacts were compared and dependencies on type of electrode and participant identified (see “Statistics” section).

All further analyses were performed only on N2 and N3 sleep epochs that were artifact-free in all four EEG derivations.

#### Analysis of Sleep Macrostructure

We compared the two single-derivation scorings by characterizing sleep-scored epochs for sensitivity (proportion of epochs scored as a particular sleep stage, which were identical in the opposite scoring), specificity (proportion of epochs not scored as a particular sleep stage, which were also not scored as this sleep stage in the opposite scoring), precision (proportion of identically scored epochs of a particular sleep stage scoring), accuracy (total proportion of identical scoring) and inter-scoring reliability (κ, Cohen’s kappa).

#### Analysis of Sleep Microstructure

We compared important characteristics of slow waves and sleep spindles between both electrode types in EEG_DrDr_ and EEG_PgPg_ (Figure 3B). Both signals were band-pass filtered between 0.5 and 4.0 Hz to automatically detect single slow waves by their negative peaks (Riedner et al., 2007). We only included slow waves in the analysis when consecutive zero-crossings were 0.25–1.0 s apart and the negative peak amplitude was greater than 37.5 μV. The duration of slow waves was determined by computing the time from the negative zero-crossing before the negative peak to the next negative zero-crossing after the negative peak. The maximum negative amplitude was the minimum amplitude of the signal during that time. The descending and ascending slope of slow waves was computed by taking the minimum and maximum of the derivative of the negative half of the signal, so the time from the negative zero-crossing before the negative peak to the positive zero-crossing after the negative peak, respectively (Figure 3B).

To automatically detect single spindles, the EEG signals were band-pass filtered between 10 and 16 Hz. The algorithm detected sleep spindles whenever an amplitude fluctuation exceeded an upper threshold that was five times higher than the average signal amplitude (Ferrarelli et al., 2007). Their start and end were detected whenever the signal dropped below a lower threshold that was 1.25 times higher than the average signal amplitude. These thresholds were suitable for detecting slow sleep spindles previously (Lustenberger et al., 2015), which is of particular importance as frontal derivations primarily show slow spindles (Cox et al., 2017). The duration of sleep spindles was calculated as the time in between the start and end of detected spindle events. The maximum amplitude was calculated as the maximum of absolute amplitude values during that time. The frequency was determined by the number of positive peaks over the duration of a spindle event. The amplitude rate was calculated by taking the sum of absolute amplitude values over the duration of a spindle event.

The agreement between EEG_DrDr_ and EEG_PgPg_ was examined using relative difference plots (Pollock et al., 1992; Giavarina, 2015) as the variability of slow waves and spindles characteristics increased as the magnitude of the measurement increased. Unlike standard Bland-Altman plots (Bland and Altman, 1999), relative difference plots depict the mean value against the ratio instead of the difference of two measurements. The average of the ratio between EEG_DrDr_ and EEG_PgPg_ described the relative bias. The relative difference value for each epoch was obtained by computing the mean characteristics in a 20 s sliding window, with a step size of 2 s, and calculating the median over all windows that covered the center of the epoch. This approach minimized the effects from characteristics that were spanning over two epochs.

Furthermore, distributions of slow-wave and spindle properties from both electrodes were compared using the overlap-index η (Pastore and Calcagnì, 2019), expressing the percent-overlap between two distributions were reported in the Supplementary Material.

#### Analysis of EEG Signal Quality

**FIGURE 4:**
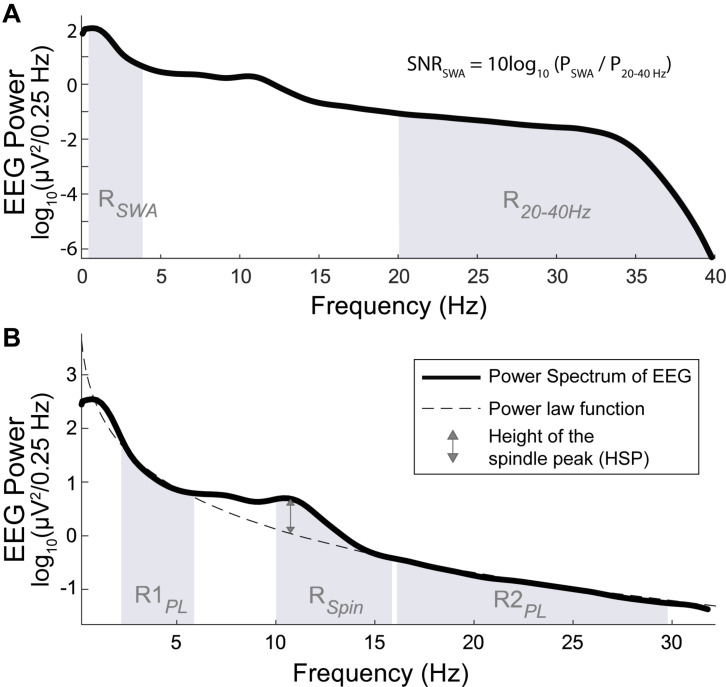
Power spectrum (bold line) across all artifact free N2 and N3 sleep epochs of one individual. (**A**, top) The SNR of SWA (SNR_SWA_) is calculated by the power ratio of the SWA frequency range R_SWA_ (shaded region between 0.5 and 4 Hz) with respect to the frequency range of no interest, R_2040 Hz_ (shaded region between 20 and 40 Hz). (**B**, bottom) The height of the spindle peak (HSP, double sided arrow) is calculated relative to the background spectrum obtained by the power law function (dashed line) fitted to the respective background power spectrum in the R1_PL_ (shaded region between 2 and 6 Hz) and R2_PL_ frequency range (shaded region between 16 and 30 Hz), excluding the 6–16 Hz range (which included R_Spin_, shaded region between 10 and 16 Hz).

For the analysis of signal quality, we compared the signal-to-noise ratio of slow-wave activity (SNR_SWA_) between EEG_DrDr_ and EEG_PgPg_. The SNR_SWA_ was determined by calculating the power ratio (dB) of the slow-wave activity frequency range R_SWA_ (0.5–4 Hz) with respect to the frequency range of no interest R_20__40 Hz_ (20–40 Hz, Figure 4A) such as

(1) SNR=SWA10log(P/SWAP)2040⁢Hz10

where P_SWA_ and P_20__40 Hz_ represented the spectral power calculated in R_SWA_ and R_20__40 Hz_, respectively. To avoid fast spindles, the low cut-off was at 20 Hz and the high cut-off was given by the previously applied band-pass filter. The agreements were compared with Bland-Altman plots.

We analyzed the height of the spindle peak (HSP) in the spectrum relative to the background spectrum in log_10_(μV^2^/0.25 Hz) units, which was adapted from the method proposed by Gottselig et al. (2002). A power law function was fitted to the power spectrum in the range of 2–6 Hz (R1_PL_) and 16–30 Hz (R2_PL_), excluding the 6–16 Hz range which contained the spindle peak frequencies (Cox et al., 2017). Very low frequencies (<2 Hz) were excluded because of their susceptibility to low frequency artifacts. We automatically localized the maximum peak within R_Spin_ (10-16 Hz). The distance between the maximum peak and its respective fitted value was determined as HSP (Figure 4B). Epochs were discarded from the HSP analysis when the frequency bin difference of the detected spindle peaks between EEG_DrDr_ and EEG_PgPg_ were greater than 2 Hz, indicating a failure of the automated maximum peak localization. The agreements were compared with Bland-Altman plots.

Furthermore, to analyze the frequency stability between signals obtained from different electrode types, we calculated the coherence between the EEG signals that were referenced to the same electrode type (EEG_DrDr_ vs. EEG_PgDr_ and EEG_DrPg_ vs. EEG_PgPg_). This analysis was possible due to the additional channel splitting and two amplifier setup. Magnitude squared coherence was calculated epoch by epoch using Welch’s averaged periodogram and shown in the range between 0 and 1 for each frequency band at a 0.25 Hz resolution.

For all participants, the PSD of EEG_DrDr_ and EEG_PgPg_, as well as the SNR_SWA_ and HSP for each derivation were calculated, visualized and reported in the Supplementary Material.

### Statistics

The testing whether the incidence of artifacts depended on the type of electrode or on the interaction between the type of electrode and the participant was performed with the Chi-Square Test, or the Fisher’s Exact Test when the number of observations was too small (Agresti, 2008).

For all Bland-Altman and relative difference analyses, we accounted for the non-constant and varying nature of spindles, slow waves, SNR_SWA_, and HSP across the night, as well as for the repeated-measures design when computing the limits of agreement (Bland and Altman, 2007). All statistical analyses were conducted in R-studio version 1.2.1335 (RStudio Team, 2018).

## Results

We included *N* = 4 participants (age range: 25.2–28.9 years, 3 male, 1 left handed, 1 ambidextrous) in the analysis. They showed a total sleep time of 5.4–9.9 h (mean = 7.45 h, *SD* = 1.98 h), a sleep onset latency of 1.7–23 min, and a high sleep efficiency (proportion of time spent asleep while in bed) between 89.8 and 98.2%. In total, we recorded 31.5 h of EEG, of which 3906 epochs (21.7 h) were spent in N2 or N3. Of those epochs, 822 epochs (21.04%) were marked with artifacts in at least one EEG derivation, resulting in 3084 artifact-free N2 and N3 epochs that went into the sleep microstructure and EEG signal quality analyses.

### EEG Artifacts

During the whole recording, EEG_DrDr_ contained 2193 (38.70%) and EEG_PgPg_ 2161 (38.14%) epochs with artifacts. In epochs scored as N2 and N3, EEG_DrDr_ had 584 (14.95%) and EEG_PgPg_ had 542 (13.88%) epochs with artifacts. The incidence of slow-sinusoidal, high-amplitude artifacts (967 windows in EEG_DrDr_, 9 windows in EEG_PgPg_) was dependent on the type of electrode [χ2(1) = 940.33, *p* <0.0001] as well as on the interaction between the type of electrode and the participant (*p* <0.0001). The incidence of very fast, sharp, abrupt artifacts (43 windows in EEG_DrDr_, 53 windows in EEG_PgPg_) was not solely dependent on the type of electrode [χ2(1) = 1.04, *p* = 0.31], but showed interactions between the type of electrode and participants (*p* = 0.0003).

### Sleep Macrostructure

**FIGURE 5:**
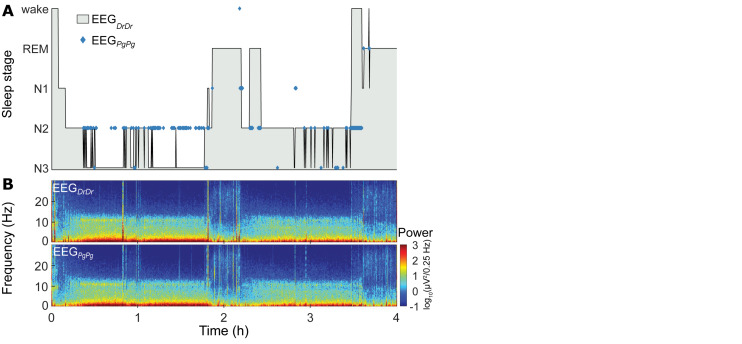
Representative sleep macrostructure for one participant. (**A**, top) Hypnogram of the single-derivation scoring based on EEG_DrDr_ of the first 4 h (two sleep cycles) of one participant. Hypnograms of the whole night of all participants can be found in Supplementary Figure S3. Blue markers indicate where the single-derivation scoring based on EEG_PgPg_ deviates from EEG_DrDr_. (**B**, bottom) Spectrogram of EEG_DrDr_ and EEG_PgPg_ derivations for the same recording.

**FIGURE 6:**
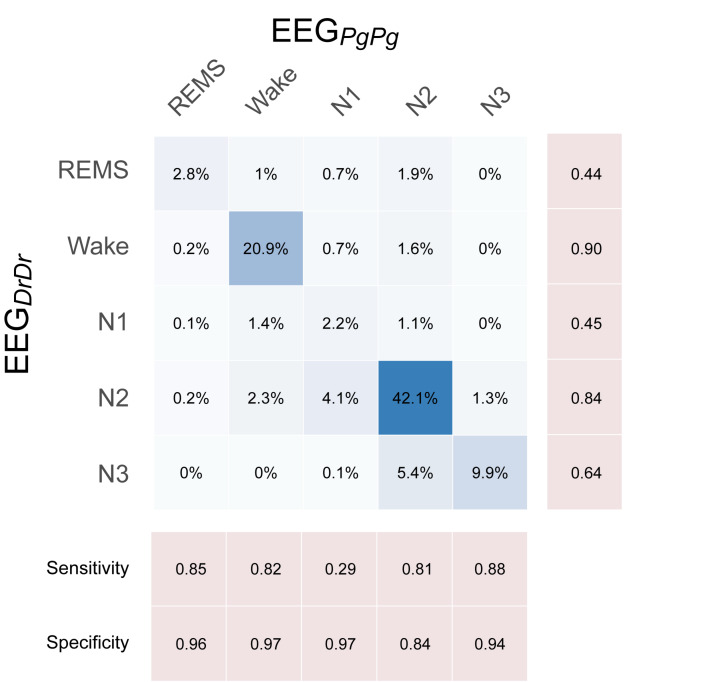
Confusion matrix showing the performance of single-derivation scoring based on EEG_DrDr_ against EEG_PgPg_, where EEG_PgPg_ served as the reference scoring. Each box contains the relative number of 20 s epochs scored as the respective sleep stage. Darker blue colors indicate higher agreement.

Sleep scorings based on the single derivations EEG_DrDr_ and EEG_PgPg_ were compared and visualized in hypnograms and spectrograms (Figure 5 and Supplementary Figure S3). Scoring between EEG_DrDr_ and EEG_PgPg_ showed an inter-scoring reliability of κ = 0.66 and an accuracy = 0.78. Only the precision and sensitivity for N1 showed poor performance (Figure 6).

### Sleep Microstructure

#### Slow Waves

The overall number of slow waves found in EEG_DrDr_ and EEG_PgPg_ was similar (+0.3% in EEG_DrDr_). The epoch to epoch comparison revealed a bias of 0.014 with limits of agreement from −2.79 to 2.82 slow waves per epoch (Table 1).

**TABLE 1:** Number of slow waves and spindles detected across all recordings and per epoch.

	**Number of slow waves**		**Number of spindles**	
	***Total***	***Per epoch median (2.5th, 97.5th centile)***	***Total***	***Per epoch median (2.5th, 97.5th centile)***
*EEG*_DrDr_	13976	3 (0, 17)	5452	1 (0, 6)
*EEG*_PgPg_	13934	3 (0, 17)	5427	1 (0, 6)

		***Mean (95% limits of agreement)***		***Mean (95% limits of agreement)***

*Difference*		0.014 (−2.79, 2.82)		0.008 (−2.34, 2.35)

**FIGURE 7:**
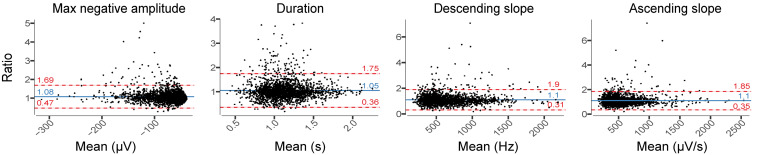
Relative difference plots showing the agreement between EEG_DrDr_ and EEG_PgPg_ for all slow-wave characteristics. For each 20 s epoch, we computed the median for each slow-wave characteristic over ten 20 s sliding window (steps of 2 s) that contained the mean value of each characteristic. Each dot represents one 20 s epoch result. The y-axis shows the ratio between the two paired measurements (EEG_DrDr_/EEG_PgPg_) and the x-axis represents the mean of these measures [(EEG_DrDr_ + EEG_PgPg_)/2]. The solid line shows the mean difference between the two paired measurements (bias, blue) and the underlying shaded area depicts the 95% CI of the bias. The limits of agreement of ratio contain 95% of measurements (dashed lines, red) and the underlying shaded red area represents the 95% CI of the limits of agreement.

Relative difference analysis of slow wave characteristics revealed among all participants a small shift of the mean difference toward larger amplitude and longer slow waves in EEG_DrDr_ recordings (Figure 7). The relative limits of agreement were 0.47–1.69 for the maximum negative amplitude, 0.36–1.75 for duration, 0.31–1.90 for descending slope, and 0.35–1.85 for ascending slope. A visual inspection of the EEG waveforms revealed that larger deviations between electrodes usually occurred from missed slow waves of smaller amplitudes or duration, attenuating the average value in one but not the other electrode because of their sparse occurrence (data not shown).

#### Spindles

The overall number of spindles was similar between EEG_DrDr_ and EEG_PgPg_ (+0.46% in EEG_DrDr_). The epoch to epoch comparison revealed a bias of −0.008 with limits of agreement from −2.34 to 2.35 spindels per epoch (Table 1).

**FIGURE 8:**
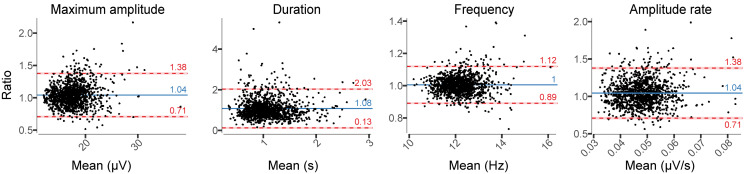
Relative difference plots showing the agreement between EEG_DrDr_ and EEG_PgPg_ for all spindle characteristics. Dots, *x*-axes, *y*-axes, solid lines, dashed lines, red bars, and blue bars can be interpreted as in Figure 7.

Relative difference analyses in spindle characteristics showed larger amplitudes and duration in EEG_DrDr_ than in EEG_PgPg_, whereas the frequency remained stable (Figure 8). The relative limits of agreement for the maximum amplitude ranged from 0.71 to 1.38, duration from 0.13 to 2.03, for the frequency from 0.89 to 1.12, and amplitude rate from 0.71 to 1.38. A visual inspection revealed for spindles that larger deviations between electrodes usually occurred from missed spindles of smaller amplitudes and duration, attenuating the average value in EEG_PgPg_.

### EEG Signal Quality

#### SNR of SWA

**FIGURE 9:**
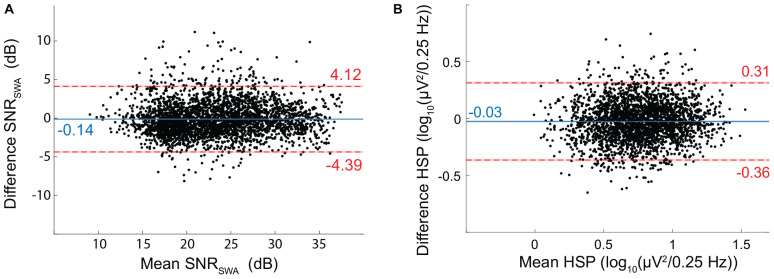
Bland-Altman for EEG Signal quality metrics. **(A)** SNR of SWA (SNR_SWA_) agreement between EEG_DrDr_ and EEG_PgPg_. **(B)** Height of the spindle peak (HSP) agreement between EEG_DrDr_ and EEG_PgPg_. The mean (*x*-axis) and difference (*y*-axis) of EEG_DrDr_ and EEG_PgPg_ were calculated for each epoch. Dots, solid lines, dashed lines, red bars, and blue bars can be interpreted as in Figure 7.

EEG_DrDr_ and EEG_PgPg_ had a mean SNR_SWA_ of 23.32 ± 5.56 dB and 23.46 ± 5.47 dB, respectively. The bias (−0.14 dB) and limits of agreement (−4.39 to 4.12 dB) revealed good agreement between the two electrode types (Figure 9).

#### Height of Spindle Peak

We discarded 397 out of the 3084 epochs (12.9%) because of inexact spindle peak detection. EEG_DrDr_ and EEG_PgPg_ had a mean HSP of 0.75 ± 0.27 and 0.77 ± 0.26 log_10_ (μV^2^/0.25 Hz), respectively. The bias (−0.03) and limits of agreement (−0.36 to 0.31) revealed a good agreement between the two electrode types (Figure 9).

#### Coherence

**FIGURE 10:**
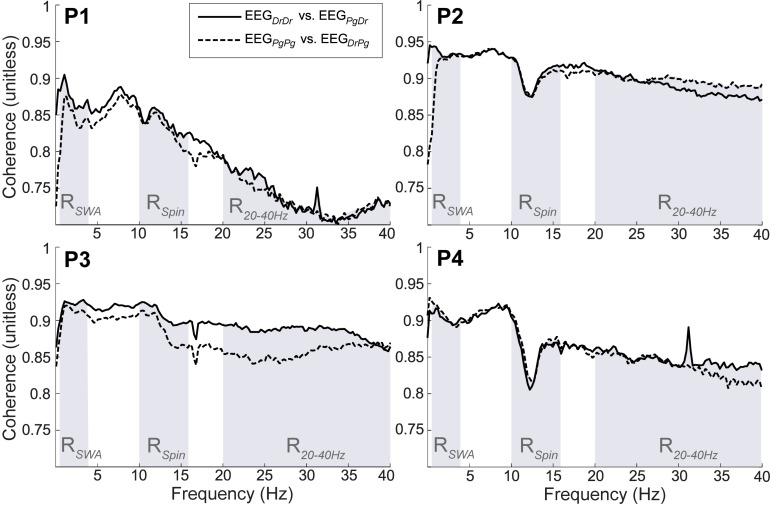
Magnitude of squared coherence between EEG_DrDr_ and EEG_PgDr_ (continuous line) and EEG_DrPg_ and EEG_PgPg_ (dashed line) for participants (P1–P4). The shaded regions represent the frequency range of slow wave activity (R_SWA_, 0.5–4 Hz), sleep spindles (R_Spin_, 10–16 Hz) and higher frequencies (R_2040 Hz_, 20–40 Hz). The maximum possible coherence is 1.

All participants showed strong coherence between EEG_DrDr_ and EEG_PgDr_ as well as EEG_PgPg_ and EEG_DrPg_ (Figure 10). The coherence coefficients were all greater than 0.70 over the total frequency range, greater than 0.80 over the slow wave range, and greater than 0.80 over the sleep spindle range. P1 had less strong coherence coefficients compared to the other participants (P2, P3, P4) in the higher frequency range (20–40 Hz), which may have been caused by high frequency artifacts present only in Dr electrodes in this participant.

## Discussion

In this study, we present a comprehensive testing protocol comparing essential features of both the macro- and, more importantly, the microstructure of sleep in EEG signals derived from two different kinds of electrodes. With this testing protocol, we evaluated whether a new generation of dry electrodes with a biocompatible, low impedance contact surface are in principal suitable for sleep EEG assessments in a proof-of-concept study. We demonstrated that the signal quality can be quantified by signal coherence, SNR of SWA, and HSP and that two types of electrodes configured in a frontal-mastoid electrode setting can be compared. Sleep specific features, such as slow waves and sleep spindles, as well as their individual characteristics, were discriminable. In addition, visual sleep scoring was performed on single EEG derivations for each of the two electrode types and did not lead to any substantial differences in the corresponding hypnograms. An increased level of artifacts in the form of slow-sinusoidal, high-amplitude artifacts was observed in the dry electrode EEG. The methodology developed for this evaluation is one of the most detailed published to date and enables an objective evaluation of sleep micro- and macrostructure characteristics obtained from dry electrodes for wearable sleep monitoring outside the lab.

Validation of electrode performance, in particular for novel wearable EEG, is challenging. Casson divided performance factors into four levels: (1) functional testing, (2) technical performance, (3) manufacturing performance, and (4) variability in performance (Casson, 2019). While level 1 and 2 investigations have been conducted previously on the electrode type of interest (Stauffer et al., 2018), our aim was to conduct a proof-of-concept study for a level 4 investigation to gain deeper insight of the technical performance for a particular application, i.e., the monitoring of sleep in a home environment. This application limits the types of tests that can be performed, e.g., the use of a head phantom is excluded (Casson, 2019). However, in such a setting, application specific features from the EEG waveform can be assessed more realistically. Former studies that investigated sleep specific use of electrodes have primarily focused on macrostructure features that characterize sleep, such as the comparison of sleep staging (Griessenberger et al., 2013; Sterr et al., 2018) and sleep timing parameters (Casson, 2019) against an established standard. In addition, the visual comparison of power spectral density (Debener et al., 2015; Stauffer et al., 2018) and/or individually selected, exemplary signal traces (Stauffer et al., 2018; Sterr et al., 2018; Shustak et al., 2019) is common. While these assessments give an overall picture of the suitability to use the wearable device for sleep monitoring, the signal quality and the suitability to assess the sleep microstructure remain unknown. This limits the findings to only basic sleep applications, but leaves the question whether the electrodes are suitable for use in research and clinical applications unanswered. We have introduced sleep microstructure specific measures to obtain a set of parameters that can be used to assess and compare in detail EEG and quality thereof. Many of these features are independent of spatial placement of electrodes and therefore suitable for comparisons under free-living conditions.

The detection of presence and characterization of shapes of sleep microstructure elements in an EEG are essential to assess the nature and quality of sleep. Our signal quality analyses clearly showed that the EEG signal quality of the electrodes is sufficient to study microstructures of brain activity during sleep. Specifically, the SNR of SWA is a measure that indicates the discriminatory power of slow waves and therefore is a good indicator for how easy it is to classify slow wave sleep. Our analyses elucidated that there is no distinct difference in bias and limits of agreement between EEG_DrDr_ and EEG_PgPg_. Slow-wave characteristics, such as their number, maximum negative amplitude, duration, or descending and ascending slope were similar in both electrodes. Larger differences between electrodes were primarily an artifact of the automated detection algorithm used for the analysis. For example, the period of slow waves was determined by the time in between two consecutive negative zero crossings of the low-pass filtered EEG signal. Occasionally, the EEG signal of one derivation marginally crossed the zero line, whereas in the other derivation the zero line was not crossed, which resulted in a large period difference. Therefore, the investigated slow wave characteristics might be more similar between the two types of electrodes than our results would suggest. Similarly, the HSP is an important sleep biomarker for quantifying the presence of spindles. Spindles are the second key electrophysiological characteristic of NREM sleep and a very sensitive feature for reduced sleep quality due to environmental, nutritional or hormonal factors (Driver et al., 1996; Borbély et al., 1999). Moreover, together with slow oscillations, they are critically involved in memory consolidation during sleep (Rasch and Born, 2013). Again, no unexpected difference in HSP between electrodes was observed. Spindles with similar characteristics could be equally identified in the Dr and Pg EEG signal. Spindle number, their maximum amplitude, frequency, and power did not show any differences between the Dr and Pg EEG derivations. The duration of spindles showed more variability between Dr and Pr EEG derivations. Similar to slow waves, spindles were represented with a slightly higher amplitude and power, as well as a longer duration in the Dr EEG derivation, especially in the recording with the most spindles.

Dry electrodes are prone to a various artifacts types (Guger et al., 2012). The electrodes showed high resistance to electrical artifacts such as electrode pops emerging from abrupt impedance changes, visually recognizable in the EEG as very fast, abrupt, sharp artifacts. However, the Dr electrodes suffered significantly more from another type of artifact, which was identified as slow-sinusoidal, high-amplitude artifacts. The amplitude and the period of such artifacts are similar to slow waves. Therefore, especially when sleep scoring is based on a single EEG derivation, those artifacts could easily be mistaken for slow waves, which biases the scorer toward scoring deep sleep. However, this bias was marginal as no significant differences in scoring deep sleep were observed. One possible source for those artifacts could have been active sweat glands. They by themselves produce slowly changing electrical potentials and release electrolytes, which can change the impedance between the electrode and the skin. Our current method for attaching the dry electrodes with skin-adhesive tape could have facilitated this activity and artifacts.

The sleep macrostructure is represented best with a hypnogram where sleep is temporally organized into wake, sleep stages N1, N2, N3, and REM sleep. Sleep scorings performed on a single EEG_DrDr_ and EEG_PgPg_ derivation showed good agreement with an overall accuracy of 0.78, which was comparable to current automatic sleep scoring algorithms using a single EEG derivation compared to expert scoring (Fiorillo et al., 2019). We can conclude that both types of investigated electrodes are suitable to determine the macrostructure of sleep. However, as no standard sleep montage was available, a comparison of the two single EEG derivation scorings to a reference scoring is missing. Future studies should compare the sleep scoring between a full polysomnography measured with Dr electrodes and a full polysomnography performed with conventional electrodes.

It is important to note that this study does not intend to serve as a validation study; the low number of participants and their generally good health status, as well as the fact that only a single night was recorded prevent any conclusions related to the performance in a clinical setting. We limited the analysis to four participants as the aim was to establish a reliable testing protocol to evaluate electrodes for advanced sleep applications and to test the general feasibility of this protocol.

Though not the primary goal of our proof-of-concept study, our results nevertheless allow some conclusions related to the ongoing technical development of the Dr electrodes. For example, at this prototype stage of the tested Dr electrodes, it remains unclear how the electrode can be reliably fixated at the desired derivations, particularly for the standard reference electrodes at the limited space behind the ears (mastoids). The fixation will need to ensure sufficient contact pressure and low movement throughout the night. We temporarily resolved this with skin-adhesive tape, which likely caused more sweating, but more importantly, reduced comfort and required additional effort during placement. A smaller reference electrode optimized for the space behind the ear would most likely increase data quality and stability.

The sleep electrode analysis could be further expanded with additional, application-specific characteristics of sleep micro- or macrostructure. As such, it would be interesting to investigate how very low-amplitude sleep phenomena, such as high-frequency oscillations are represented in the EEG signal collected with dry electrodes. However, this is rarely assessed in surface electrodes, but more often in intracranial electrode recordings. Furthermore, sleep timing parameters characterizing sleep behavior that is important for clinical use, such as total sleep time, sleep onset latency or wake after sleep onset, could be added to the comparison when the focus of the application is of diagnostic nature. These statistics should only be assessed and compared when a high number of participants is available, as the inter-subject variability is high. To complement the technical performance evaluation in completely free-living conditions in future studies, it would be crucial to evaluate the usability and human-device interaction specific to electrode application and resulting quality. For example, self-administration, changing environmental conditions such as humidity and temperature as well as the variation of external noise sources specific to individual’s bedrooms would require studies with much larger population sizes. This evaluation step was currently not possible as the electrodes were not yet integrated into a single system.

A reliable detection of sleep EEG markers to characterize sleep micro- and macrostructure is essential for sleep research and many clinical applications. However, detailed clinical and scientific sleep assessments are usually performed in a lab environment. This approach is mainly due to the need for high-quality sleep EEG to assess sleep-specific neurophysiological events and the high level of manual configurations needed to operate such systems. Needless to say that such a procedure is cumbersome for both the participant and researcher and, as primarily good sleepers are selected for studies, leads to results biased toward single nights with good sleep characteristics. With dry electrode technologies continuously improving and wearable EEG system becoming more and more available, sleep assessments will likely move from the sleep lab to a home setting where multiple consecutive nights can be assessed in a familiar setting. This allows the long-term recording of natural sleep behavior in more representative populations, which is of high relevance for clinical populations with an increased risk for a sleep disorder. Critical requirements for future electrodes will be that they are reusable, easy to apply, and capable of being combined with low-cost mobile EEG amplifier systems.

## Conclusion

This study provides a carefully conceptualized testing protocol to not only evaluate the macro-, but also the microstructure of sleep in EEG signals derived from two different kinds of electrodes. Our extensive comparison of the performance of novel dry electrodes to pre-gelled electrodes in four sleep EEG recordings obtained in a home setting shows the potential of dry electrodes for sleep EEG assessments. Both electrodes reliably recorded slow waves and sleep spindles, which are features of specific interest in sleep research. The signal-to-noise ratio was similar in dry electrodes compared to pre-gelled electrodes. The proposed testing paradigms highlighted similarities and differences between electrode types and can be applied on sleep EEG collected from both the lab and the home.

## Data Availability Statement

The dataset containing the four nightly recordings used in this study are publically available under doi: 10.3929/ethz-b-000416415.

## Ethics Statement

The studies involving human participants were reviewed and approved the by Ethikkommission der ETH Zürich (EK ETH 2017-N-67). The participants provided their written informed consent to participate in this study.

## Author Contributions

All authors conceived and designed the project. KC and WK prepared the hardware and software for running the experiments. KC and SL conducted the experiments. KC, SL, and WK performed the analysis of the data. KC, SL, LT, WK, and RH drafted the manuscript. SL, LT, RH, and WK revised and approved the final version of the manuscript.

## Conflict of Interest

The authors declare that the research was conducted in the absence of any commercial or financial relationships that could be construed as a potential conflict of interest.
