# Supplementary material for: A Protocol for Comparing Dry and Wet EEG Electrodes During Sleep
Source: Front Neurosci. 2020 Jun 17;14:586. doi: 10.3389/fnins.2020.00586 (PMC7313551; doi:10.3389/fnins.2020.00586)
Supplement: Supplementary file 1 [file Data_Sheet_1.pdf]

A protocol for comparing dry and wet EEG electrodes during sleep

Supplementary Material

1 Supplementary Tables

Table S1. List of acronyms.

Acronym	Meaning
EEG	Electroencephalogram
Dr	Dry electrode
Pg	Pre-gelled electrode
N1, N2 and N3	Non-rapid-eye-movement sleep stage 1, 2 and 3
REMS	Rapid-eye-movement sleep
EEG_{Dr}	EEG measured using Dr
EEG_{Pg}	EEG measured using Pg
EEG_{DrDr}	EEG_{Dr} measured while grounded and referenced to Dr
EEG_{PgPg}	EEG_{Pg} measured while grounded and referenced to Pg

2 Supplementary Figures

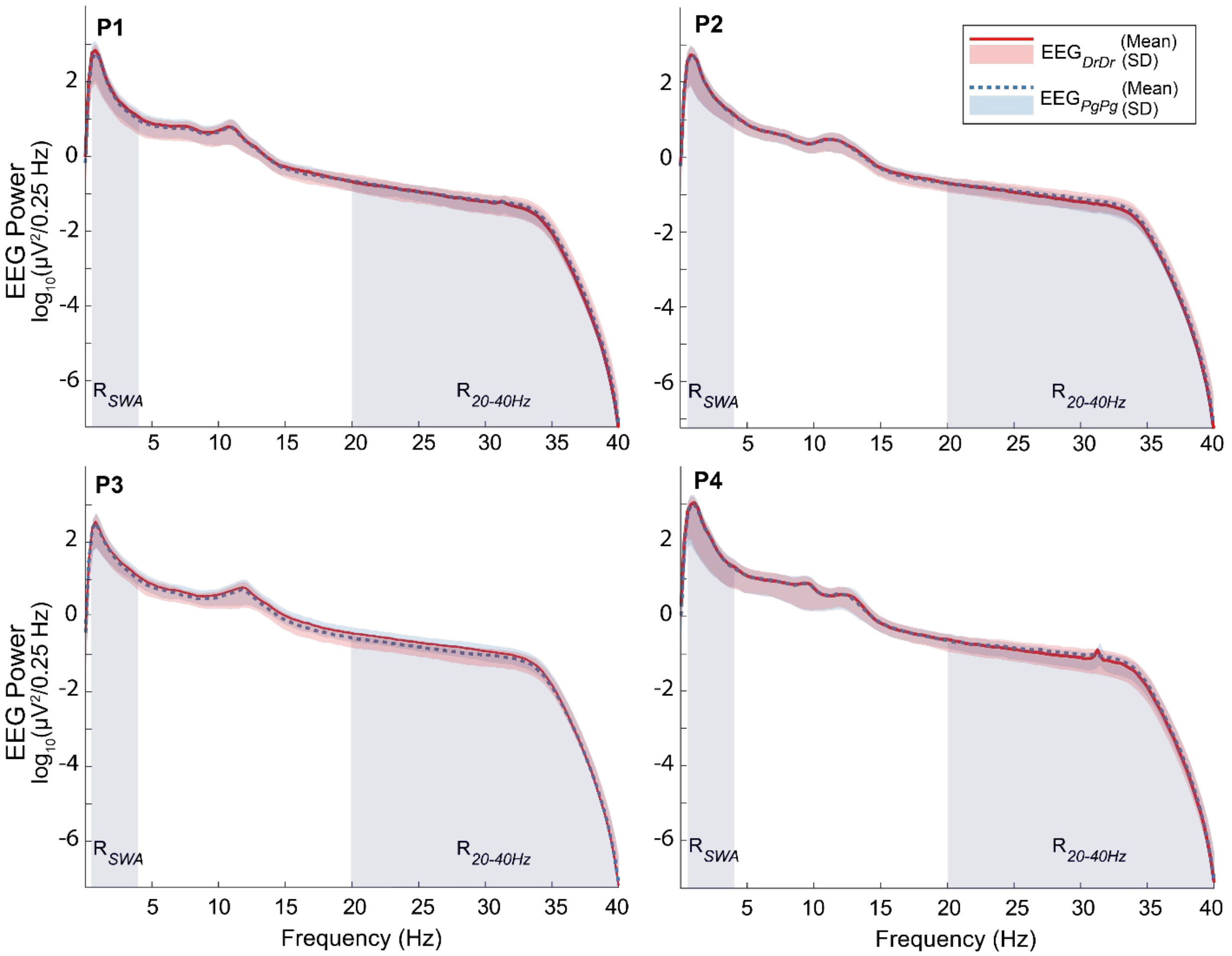

Figure S1. EEG power spectrum to calculate the signal-to-noise ratio (SNR) of slow wave activity (SWA) SNR_{SWA} of the four participants (P1–P4). The graph shows the power spectrum of EEG_{DrDr} (red continuous line) and EEG_{PgPg} (blue dotted line). The shaded region around the power spectrum represents the SD of EEG_{DrDr} (red shaded region) and EEG_{PgPg} (blue shaded region) across all artifact-free N2 and N3 sleep epochs of one overnight recording. The SNR_{SWA} is calculated by the power ratio of the SWA frequency range R_{SWA} with respect to the frequency range of no interest, $R_{2040\ Hz}$.

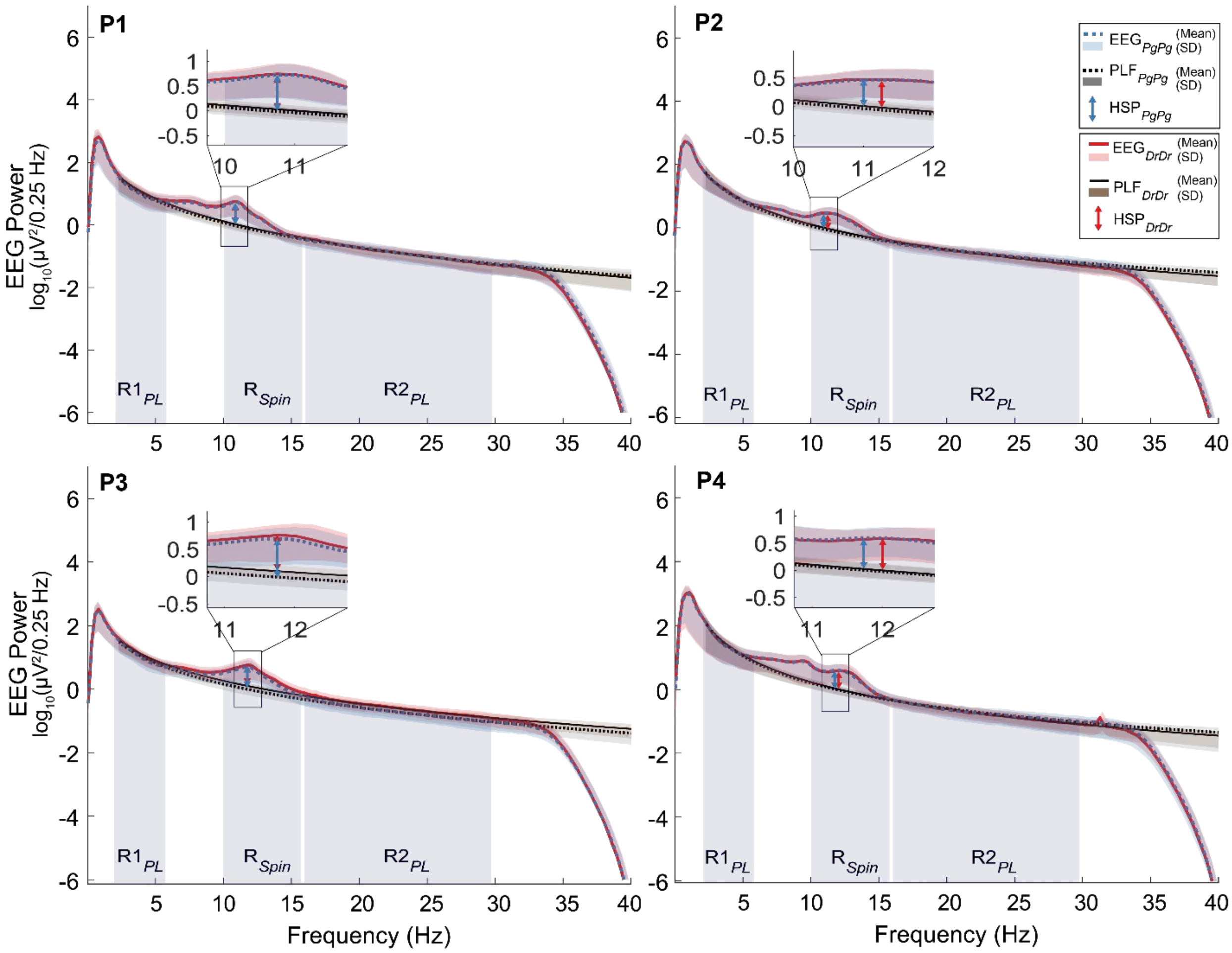

Figure S2. Height of the spindle peak (HSP) for four participants (P1–P4). The graph shows the mean power spectrum of EEG_{DrDr} (red continuous line) and EEG_{PgPg} (blue dotted line). The shaded region around the power spectrum represents the SD of EEG_{DrDr} (red shaded region) and EEG_{PgPg} (blue shaded region) across all artifact-free N2 and N3 sleep epochs of one overnight recording. The power law function (PLF) of EEG_{DrDr} (PLF_{DrDr}) and EEG_{PgPg} (PLF_{PgPg}) was each fitted to the respective background power spectrum in the $R1_{PL}$ (shaded region between 2–6 Hz) and $R2_{PL}$ frequency range (shaded region between 16–30 Hz), excluding R_{Spin} (shaded region between 10–16 Hz). The graph shows the mean PLF_{DrDr} (black continuous line), SD PLF_{DrDr} (light brown shaded region), mean PLF_{PgPg} (black dotted line) and SD PLF_{PgPg} (dark gray shaded region). HSP of EEG_{DrDr} (HSP_{DrDr}, red double headed arrow) and EEG_{PgPg} (HSP_{PgPg}, blue double headed arrow) is each determined by calculating the difference between the fitted value and the measured value at the respective frequency. The difference was calculated for all possible frequencies within R_{Spin}. The maximum difference was determined as HSP. Due to the limitation of automatic spindle peak detection, the following epochs were discarded for HSP analysis when the frequency bin difference of the detected spindle peaks between EEG_{DrDr} and EEG_{PgPg} was greater than 2 Hz: **P1**, 125 out of 700 epochs (17.86%); **P2**, 90 out of 689 epochs (13.06%); **P3**, 77 out of 848 epochs (9.08%); and **P4**, 105 out of 847 epochs (12.40%).

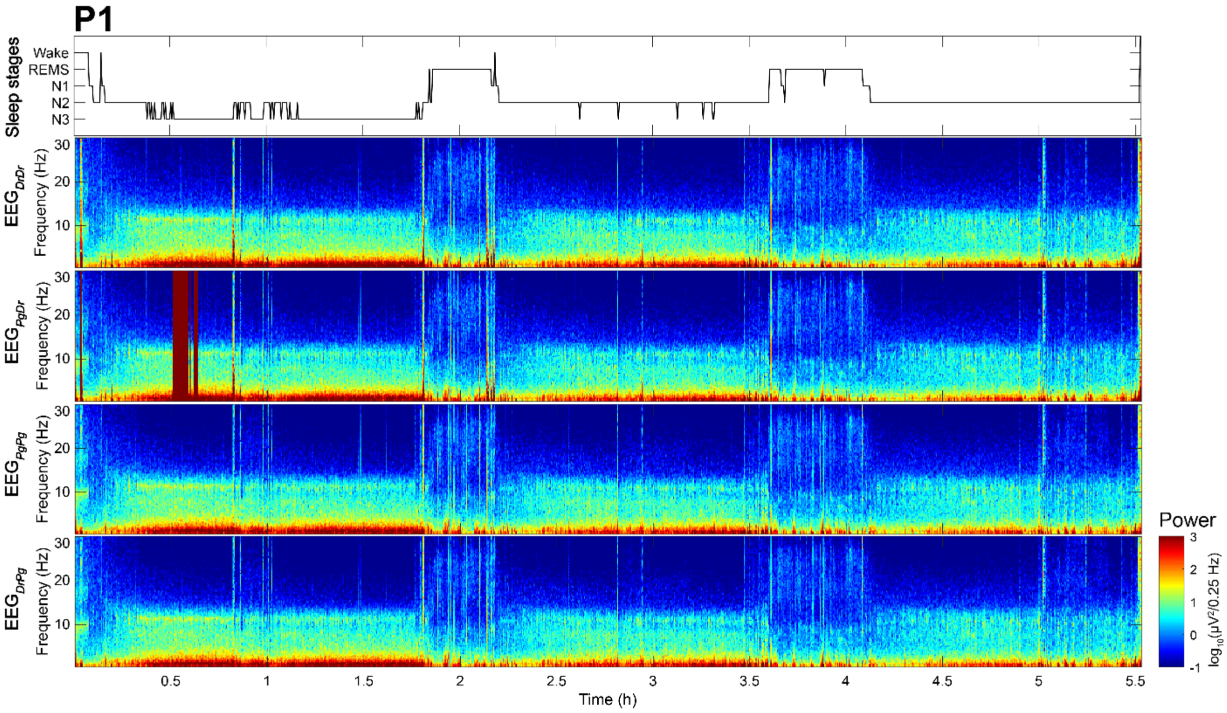
P1
Sleep stages
Wake
REMS
N1
N2
N3
EEG_DrDr
EEG_PgDr
EEG_PgPg
EEG_DrPg
Frequency (Hz)
Power
log10(μV²/0.25 Hz)
Time (h)

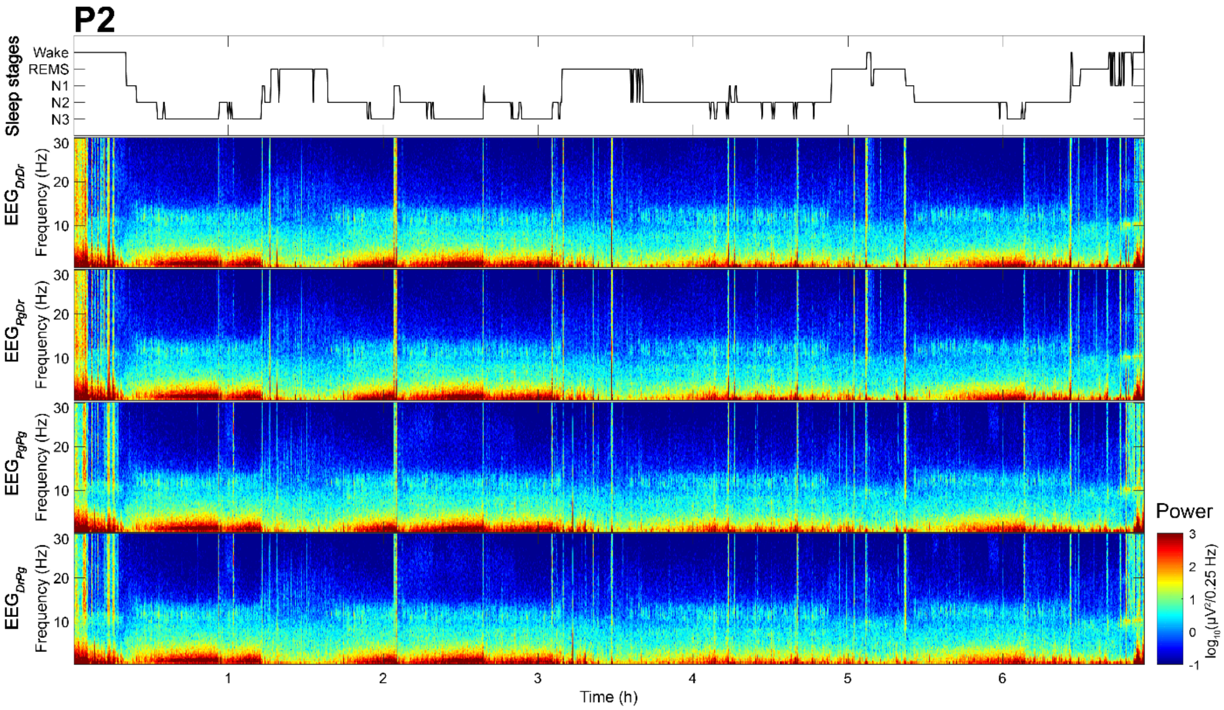
P2
Sleep stages
Wake
REMS
N1
N2
N3
EEG_DrDr
EEG_PgDr
EEG_PgPg
EEG_DrPg
Frequency (Hz)
Power
log10(μV²/0.25 Hz)
Time (h)

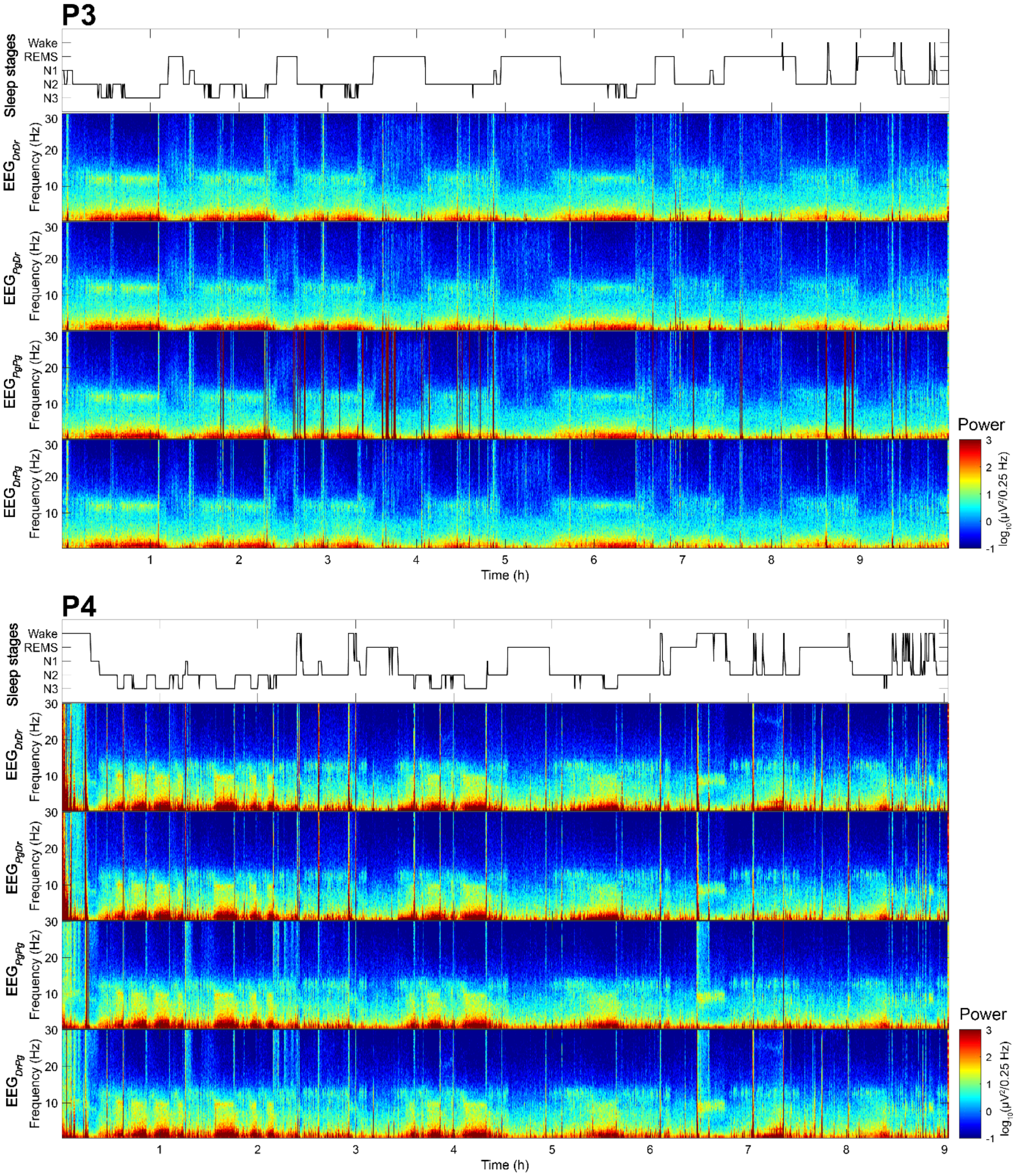

Figure S3. Hypnograms (top graph) obtained from all available EEG, EOG and EMG channels and the spectrograms of EEG$_{DrDr}$, EEG$_{PgDr}$, EEG$_{PgPg}$ and EEG$_{DrPg}$ (bottom graphs) for each participant (P1-P4). The x-axis represents time divided into sleep epochs (20 s per epoch). The y-axis of the hypnogram represent sleep stages (Wake, REMS, N1, N2 and N3) and the y-axis of the spectrogram represents frequency (in Hz). The color represents the spectral power density.

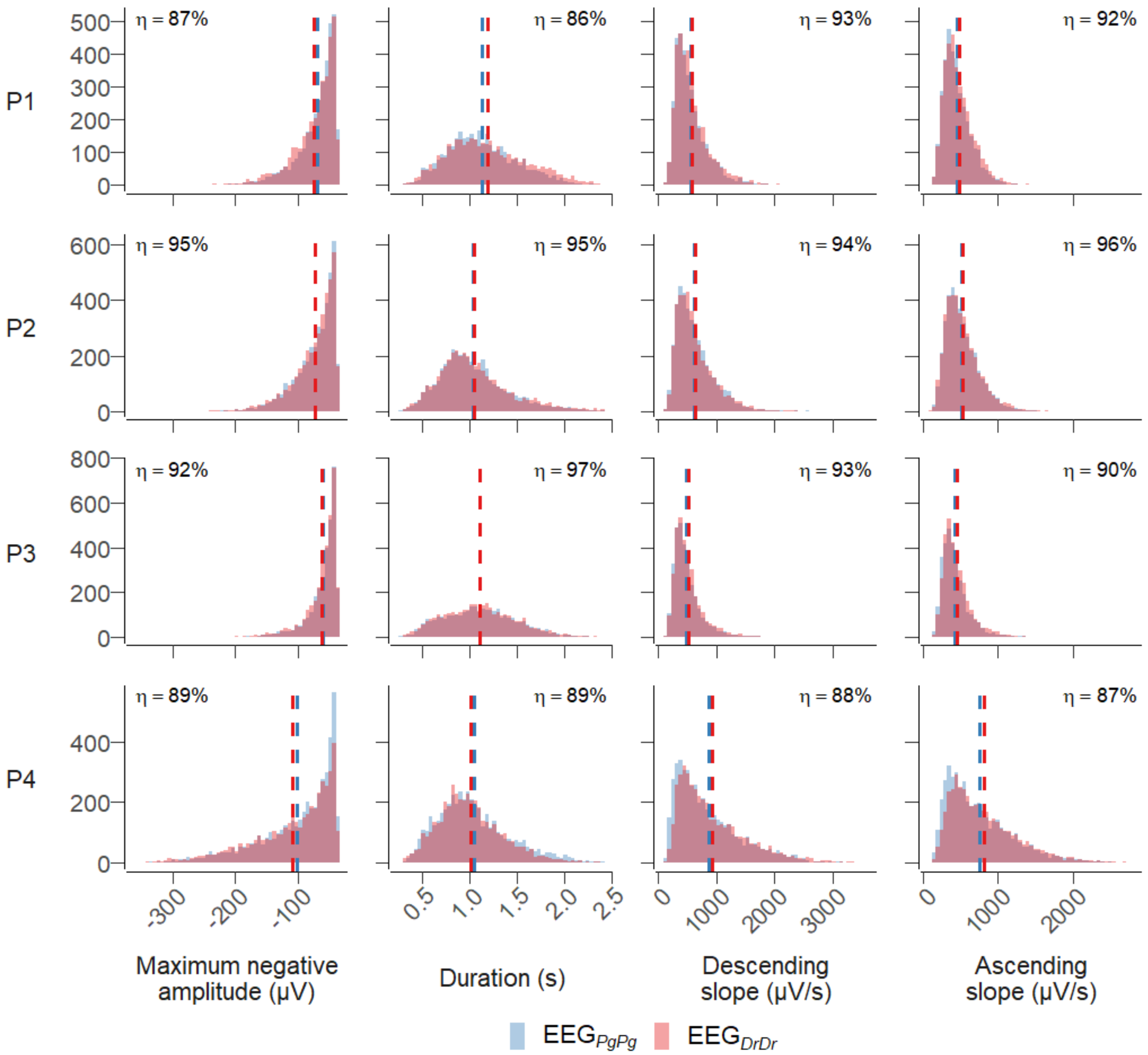

Figure S4. Histograms of slow-wave properties for each participant (P1–P4). Slow waves were detected in both EEG_{DrDr} (blue) and EEG_{PgPg} (red). For illustration purposes, we do not show extreme values deviating more than ± 3.5 SD (this concerned 0.0005% of the data, determined on log-transformed data if the data is skewed). Dashed lines indicate the mean value. The overlap-index η shows percent-overlap between the two distributions. All distributions of slow-wave properties measured with both electrodes overlap nearly completely.

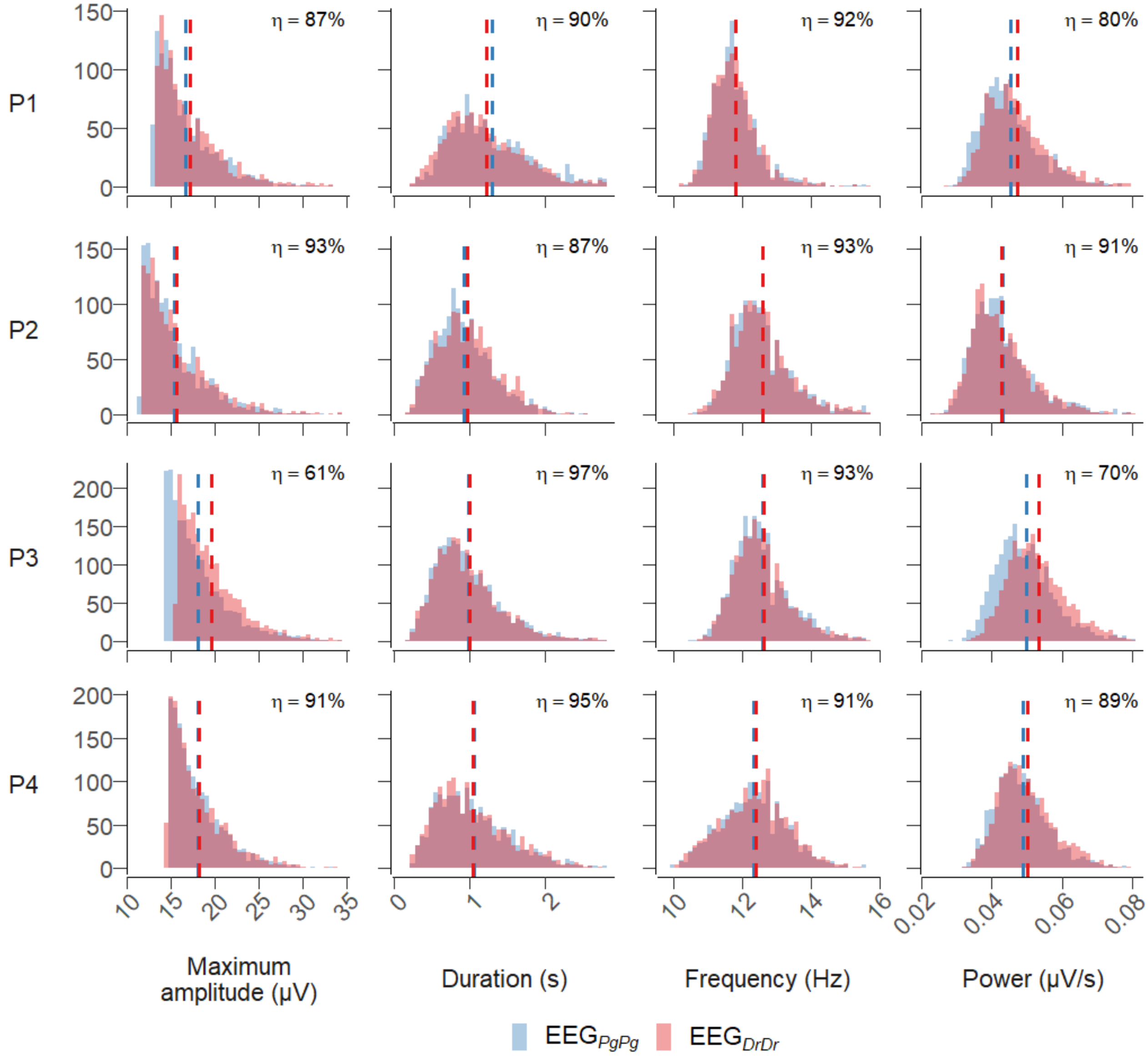

Figure S5. Histograms of spindle properties for each participant (P1–P4). Spindles were detected in both EEG_{DrDr} (blue) and EEG_{PgPg} (red). For illustration purposes, we do not show extreme values deviating more than ± 3.5 SD (this concerned 0.0054% of the data, determined on log-transformed data if the data is skewed). The distributions of spindle duration and spindle frequency of both electrodes overlap almost completely. Dashed lines indicate the mean value. The overlap-index η shows percent-overlap between the two distributions. The distribution of amplitude-based spindle properties is shifted towards higher values in EEG_{DrDr}.

3 Supplementary Code (Filter code)

We provide the Matlab code used for filtering the EEG, EMG, and EOG signal for all conducted analyses. The function `pop_eegfiltnew()` was part of the EEGLAB toolbox (version 14_1_2b, https://sccn.ucsd.edu/eeglab).

For EEG signal quality analysis, the EEG signal was notch-filtered at 50 Hz and band-pass filtered between 0.5–40 Hz using the following code:

```
fs = 250;             % Sampling rate

%% 50 Hz notch filter %%
wo = 50/(fs/2);
bw = wo/15;
[num,den] = iirnotch(wo,bw);
EEG = filter(num,den,EEG);

%% 0.5-40 Hz bandpass filter %%
 bpFilt = designfilt('bandpassiir','FilterOrder',24, ...
     'StopbandFrequency1',0.375,'StopbandFrequency2',40, ...
     'SampleRate',250,'DesignMethod','cheby2','StopbandAttenuation',60);

EEG = filter(bpFilt, EEG);
```

For sleep scoring, the EEG signal was band-pass filtered between 0.5–4 Hz, the EMG signal was band-pass filtered between 20–40 Hz, and the EOG signal was band-pass filtered between 0.3–4 Hz using the following code:

```
EEG = pop_eegfiltnew(EEG, 0.5, [],  [], false, [], 0);
EEG = pop_eegfiltnew(EEG, [],  40, 170, false, [], 0);

EMG = pop_eegfiltnew(EMG, 20,  [],  [], false, [], 0);
EMG = pop_eegfiltnew(EMG, [],  40, 170, false, [], 0);

EOG = pop_eegfiltnew(EOG, 0.3, [],  [], false, [], 0);
EOG = pop_eegfiltnew(EOG, [],  40, 170, false, [], 0);
```

For the detection of slow waves, the EEG signal was band-pass filtered between 0.5–4 Hz using the following code:

```
EEG = pop_eegfiltnew(EEG, 0.5, [], 3300,  false, [], 0);
EEG = pop_eegfiltnew(EEG, [],   4,  830,  false, [], 0);
```

For the detection of spindles, the EEG signal was band-pass filtered between 10–16 Hz using the following code:

```
bpFilt = designfilt('bandpassiir', ...
           'PassbandFrequency1',10, 'PassbandFrequency2',16, ...
           'StopbandFrequency1', 5, 'StopbandFrequency2', 32, ...
           'StopbandAttenuation1', 80, 'StopbandAttenuation2', 80, ...
           'PassbandRipple', 3, ...
           'SampleRate', 250, ...
           'DesignMethod', 'cheby2');
EEG = filtfilt(bpFilt, EEG);
```
